# Implementing national care guidelines in local authorities in England and Wales: a theory-of-change

**DOI:** 10.1186/s12913-024-11707-4

**Published:** 2024-10-12

**Authors:** Annette Bauer, Annette Boaz, Erica Breuer, Ties Hoomans, Sarah Jasim, Martin Knapp, Joaquín Mayorga Camus, Juliette Malley

**Affiliations:** 1 Care Policy and Evaluation Centre, London School of Economics and Political Science, London, WC2A 2AE UK; 2 The Policy Institute, King’s College London, Strand, London, WC2R 2LS UK; 3 School of Health, Medicine and Wellbeing, University of Newcastle, University Drive, Callaghan, NSW 2308 Australia

**Keywords:** Theory-of-change, Participatory, Theory-informed, Implementation, Evaluation, National guidelines, Social care, Long-term care

## Abstract

**Background:**

The delivery of high-quality services in chronically underfunded social or long-term care systems is a major challenge internationally. National guidelines, developed by the National Institute for Health and Care Excellence, set out how local authorities in England and Wales should fund and provide care based on best available evidence. Theoretical and participatory approaches can usefully inform the design and evaluation of implementation strategies for guidelines. The aim of the study is to develop a Theory-of-Change for how the implementation of these guidelines is expected to lead to impacts from a local authority perspective.

**Methods:**

As part of a comparative case study (The ‘Valuing Care Guidelines’ study; February 2022 to April 2024) with three local authority sites in England and Wales, we involved altogether 17 participants in two Theory-of-Change online workshops per site, each of 2 hours. Additional data gathered from the same participants as part of the overall study were used to conceptualise and enrich information from the workshops.

**Results:**

Participants described the Theory-of-Change map as follows: A wide range of activities (categorised in stages of ‘pre-implementation’, ‘implementation’, ‘sustainment and scaling’) and skills were required to implement guidelines, and achieve long-term organisational sustainability and service delivery outcomes, leading to final impacts for service users and carers. Participants described a co-creation implementation model, led by ‘Implementation Support Practitioners’, who utilised relational skills to achieve motivation, trust, and confidence at different organisational levels, addressing contextual barriers such as inadequate staffing, lack of resources and of organisational support systems. Consistent use of guidelines by frontline staff could only be achieved if the value of guideline implementation was promoted widely, and if consideration was given to the roles of stakeholders, such as the inspection body, local health care providers, users and carers.

**Conclusions:**

Our study is the first to investigate the implementation of national social care guidelines by local authorities in England and Wales. It generates insights that can guide implementation practice as well as inform the evaluation of future implementation strategies.

**Supplementary Information:**

The online version contains supplementary material available at 10.1186/s12913-024-11707-4.

## Background

National guidelines, by providing evidence-informed recommendations about good practice, seek to bridge the gap between research and practice in health, social care and other areas. Whilst the implementation of clinical guidelines in healthcare, alongside other vehicles to achieve evidence-based practice, has been the subject of many evaluations [1, 2], little is known about how public and non-public bodies implement national guidelines in social care (called long-term care in many countries) and what outcomes are achieved [3]. An international review of studies investigating drivers of and barriers to guideline implementation in long-term nursing care found that inadequate staffing, lack of resources and of organisational support systems often prevent guideline implementation [4]. Without knowledge about how guidelines can be implemented, the resources spent on developing them are likely to be wasted [5]. The aim of the study is to map out a pathway about how the implementation of care guidelines is expected to lead to impacts from a local authority perspective and provide insights into how implementation might be best designed and evaluated.

### Evidence-based practice and role of guidelines

In the UK, social care provides essential care, safeguarding, protection, and support for people with various needs, including those related to disability, old age and poverty. Services, provided by local authorities or independent providers, are chronically under-resourced, leading to substantial unmet needs [6]. For example, 2.6 million older people in England are estimated to have unmet care needs, substantially limiting their functional abilities and health [7, 8]. Staff recruitment and retention are key challenges facing the sector, which is known for poor working conditions including low staff pay and reflected in high staff turnover [9]. The Care Quality Commission, which is the national inspection body, rated 10% of social care as ‘inadequate’ in safeguarding and legal protection [10]. Similar issues prevail in most other countries, raising questions about how to deliver good quality care to everyone who needs it [8]. In response to this identified need for more effective and accountable practice, the English and Welsh government have promoted policies around evidence-based practice [11, 12], including the development of guidelines [13].

Since 2012, the National Institute for Health and Care Excellence (NICE), an executive body of the Department of Health and Social Care traditionally responsible for health technology assessments and clinical guidelines, has been responsible for publishing national guidelines in social care. Developed systematically in consultation with professionals and public representatives, these guidelines incorporate the findings from published studies, expert testimonies, and legislation, and provide a large number of recommendations on care practices and principles [14–16]. Their aim is to assist practitioners, managers, and commissioners in their decisions about delivering or funding care to different populations in specific circumstances [14].

### Guideline implementation and evaluation: Learnings from clinical care

In clinical care, where guidelines have been developed for decades, the many implementation barriers at individual, professional, and organisational or system levels, which can vary by topics and contents of individual guidelines, have been studied extensively [1, 17]. Multi-facetted implementation strategies that target the most important contextual barriers have been found to be more effective than single strategies [18, 19].

However, analysing the findings from 86 systematic reviews of evaluations of implementation strategies, Boaz et al. [2] conclude that “we might have gone as far as we can in understanding the implementation of evidence through systematic reviews of single and multi-faceted interventions”. Instead, participatory, and theory-informed approaches to both guideline implementation and evaluation have been suggested as a way forward to generate a contextualised and practice-relevant understanding of implementation strategies [4, 20].

### Evaluation of guideline implementation: theory-of-change method

Using participatory, theory-informed approaches, the implementation of guidelines can be investigated by focusing on how implementation strategies are expected to work, as assessed by stakeholders [21, 22]. This knowledge can help to optimise implementation processes and resources by identifying factors likely to influence outcomes, as well as inform the choice of outcome measures for an evaluation [23]. One such method is the ‘Theory of Change’ (ToC) approach, which involves stakeholders in establishing the intended impact of a programme, and mapping out how this can be achieved through a sequence of short- and long-term outcomes [23, 24]. By applying a backwards-to-forward logic, it draws out the potential causal pathways and multiple linkages between activities and outcomes, and their relationship to final impact, which are visualised in a diagram, the ‘ToC map’ [24]. The method is useful in social care due to its flexibility and focus on stakeholder inputs [25].

This paper presents our research on the local implementation of NICE guidelines by local authorities in England and Wales. Whilst the focus is on social care, the investigation is likely to be relevant to other areas of non-clinical care.

## Method

### Overview

Using the ToC method, we developed hypothetical pathways illustrating how local authorities implement NICE guidelines and the expected outcomes. This was part of a participatory, theory-informed case study (‘The Valuing Care Guidelines’ study) involving three local authority sites in England and Wales from February 2022 to April 2024. The aim of the ‘Valuing Care Guidance study’ was to assess the processes, costs and consequences of implementing guidelines in the sites, and the ToC method was developed as a conceptual framework. We gathered primary data through two online workshops per site, each lasting about 2 hours, attended by altogether 17 local authority managers (7 in site 1; 4 in site 2; 6 in site 3) responsible for and involved in implementing the guidelines. Whilst we had planned for conducting the workshops either online or in person, participants in the sites preferred workshops to take place online, as this fitted better with their work routines and for some reflected how they organised meetings since the Covid pandemic. Additional data gathered from the same individuals as part of the overall study were used to conceptualise and enrich the information from the workshops. Sources included conversations with individuals about their roles and the work they were doing, as well as templates (= activity diaries) for capturing detailed data on what individuals did, for what purpose when and for how long. Whilst all participants were approached for additional data, this was done in group emails and often responses were coordinated by one or two individuals in each site. This followed their routine way of working and communicating with each other.

Informed by Leeman et al. [26], we categorised activities into three stages: pre-implementation (creating the conditions for implementing guidelines), implementation (conducting or monitoring actions to increase adherence to guidelines), and sustainment (sharing implementation learnings within the organisation, regionally or nationally). To help with the interpretation of findings, as part of a knowledge mobilisation approach recommended by the funder of the study [27], we consulted both those with lived experience and professional experts, including NICE implementation consultants or facilitators, local authority managers experienced in implementing guidelines, social care policy makers and advisors, and public representatives involved in NICE guideline development processes.

We report our findings on local guideline implementation using the ‘Checklist for reporting ToC in Public Health Interventions’ (Breuer et al., 2016), with some adaptation based on a recent review [28]. For the completed checklist and its explanation, see the Supplementary file. Additional analyses, including those on implementation costing and methodology. This will be reported separately as part of the overall study.

### Human ethics and consent to participate declaration

The study received ethics approval from the ethics committee of the London School of Economics and Political Science and Health Research Authority (22/PR/07450). All human participants provided verbal and written consent to participate in the study and workshops.

### Study settings and participants

The three local authority sites were selected through a hybrid purposive-convenience sampling approach [29], whereby we utilised our professional networks (e.g., NICE’s team responsible for supporting the implementation of guidelines) to identify sites and participants with rich expertise in guideline implementation. The sites were comparable with regards to the guideline topics implemented and proportions of people receiving adult social care; they varied in the quality and governance arrangements for guideline implementation, type of organisation and levels of adult social care needs (see also Table 1). All sites had worked with the team at NICE that support the implementation of guidelines in the past and for two sites this was an ongoing relationship.

**Table 1:** Implementation context for guidance implementation in adult social care, for each local authority

	Site 1 (England)	Site 2 (Wales)	Site 3 (England)
**Guideline topics implemented prioritised in study**			
	NG189 Safeguarding adults in care homes NG108 Decision making and mental capacity NG86 Improving the experience of care and support for people using adult social care services	NG108 Decision-making and mental capacity & self neglect guidance *Various guideline topics identified in response to practice issues*	NG86 Improving the experience of care and support for people using adult social care services NG43 Transition from children’s to adults’ services
**Quality improvement responsibilities **			
Groups responsible for strategic oversight of guidance implementation	Adult Safeguarding Board Adult Social Care Policy, Procedure, Guidance and Public Information Group (short: Policy Working Group)	Adult Safeguarding Board Safeguarding Board’s Policy Practice & Procedure Management Group	Adult Safeguarding Board Quality and Governance Group Adult Services
Operational groups for guidance implementation	Direct Working Resource Group	Self-neglect Task & finish Group Consultant Social Worker Group	/
**Characteristics of local authority and adult social care population **			
Type of local authority (Metropolitan councils are responsible for all services, whereas county councils are not responsible for services that are managed at city or district levels, such as recycling).	Metropolitan district	County	Unitary, In-house provider
Net expenditure adult social care, £ millions	110.8	80.1	114.4
Number of employees at local authority	4,800	6,500	5,000
Number of residents	345,300	142,300	320,600
Percent of population aged 65+	13%	21.5%	25.1%
Number of adults receiving adult social care (in % of residents)	4,529 (1.3%)	2,150 (1.5%)	5,000 (1.6%)
Proportion of people with daily life limitations / adult social care needs	18.4%	24.6%	19.6%
Number of registered care homes	78	55	70
Proportion small areas in most deprived 10% nationally (employment/ income)	10.8%/15.9%	41.8%/23.1%	15.7%/10.7%

Authors’ own estimations based on Office for National Statistics (ONS) data, StatsWales, and available local sites information

Participants included those involved in NICE guideline implementation at strategic and/or operational level. They were identified with help from our main site contacts, and included what has been described in the literature as ‘Implementation Support Practitioners’ [29]. Main contacts had roles such as Principal Social Workers (in England), Consultant Social Workers (in Wales), or Quality improvement Managers or Service or Team Leads. They had specific policy and quality improvement roles, covering social work or adult social care services, which were either set out in their job descriptions or were shaped over time through professional development in agreement with (other) senior managers or directors. They identified other workshop participants based on the role they had taken on in supporting the implementation of the above-mentioned NICE guidelines, either strategically or operationally. Participants had job roles that sought to support the quality of services and develop other staff. Table 2 provides information about the procedure and participants who participated in the workshops at each site. There were some changes in main contacts and participants over the course of the study due to illness, retirement, other emerging priorities or obligations, and changes in the organisational structure.

**Table 2:** Workshop procedure and participants at ToC workshops at three local authority sites

**Site 1**	**Site 2**	**Site 3**
**Workshop details (timeline)**		
1st workshop 24/04/2023 2nd workshop 02/06/2023	1st workshop 28/02/2023 2nd workshop 14/03/2023	1st workshop 04/08/2022 2nd workshop 14/03/2023
**Main contacts for organising ToC workshops**		
Principal Social Worker	Consultant Social Workers	Principal Social Worker & Senior Quality Improvement and Assurance Manager
**Participants of ToC workshops**		
*N* = 7	*N* = 4	*N* = 6
Principal Social Worker (1st and 2nd workshop) Joint Clinical Quality Lead for Care Provision Integrated Care Board (1st workshop) Quality Assurance Manager (1st workshop) Safeguarding Adult Coordinator (1st and 2nd workshop) Senior Practitioner Social Worker (2nd workshop) Carers and Engagement Lead (1st and 2nd workshop) Practice Development Social Worker (1st and 2nd workshop)	Policy Officer Safeguarding: Adults and Child Service and Quality Assurance (1st and 2nd workshop) Consultant Quality, Performance & Practice (1st workshop) Consultant Social Worker (1st and 2nd workshop) Quality, Performance and Practice Manager	Principal Social Worker (1st and 2nd workshop) Senior Quality Improvement and Assurance Manager (2nd workshop) Senior Manager Adult Services (1st and 2nd workshop) Clinical Lead Occupational Therapist (1st and 2nd workshop) Service Manager Adult Services/ Shared Lives (1st and 2nd workshop) Operational Manager Social Work, Learning and Development (1st workshop)

### Procedure and data analysis

Before the workshops, participants received information about the study, the ToC method, the structure and content of the workshops and a consent form. Workshops included introductions about the project and the ToC method and facilitated discussions regarding past, current, or planned local activities to implement guidelines, as well the implementation problem, intervention impacts and outcomes. We used ‘Mural’ online graphic collaboration software [30] to draft and revise site-specific ToC maps, resolving conflicts or disagreements through open and respectful communication, in which all perspectives were first validated and then responses were brought back to the purpose of the workshop before deciding about group priorities and relevance to the ToC map. After the first round of workshops, the ToC maps were developed further by the research team, presented to the participants in the second workshops and then iteratively refined by the researchers using additional data from one-on-one or group meetings that we organised as part of the overall study and that covered discussions with participants about several topics relevant to the research, including about the ToC maps. We also summarised the workshops in each site with a brief report.

After developing the site-specific ToC maps, we created an overarching ToC map that covered all three sites. First, we used the existing structure of the site-specific ToC maps, which included the different stages of implementation, outcomes and impacts of implementation, to structure the overarching ToC map. Second, we grouped and summarised key outcomes and mapped these onto the existing structure of the overarching ToC map. Third, we grouped the types of implementation activities and mapped these onto the existing ToC map.

## Results

**Fig. 1:**
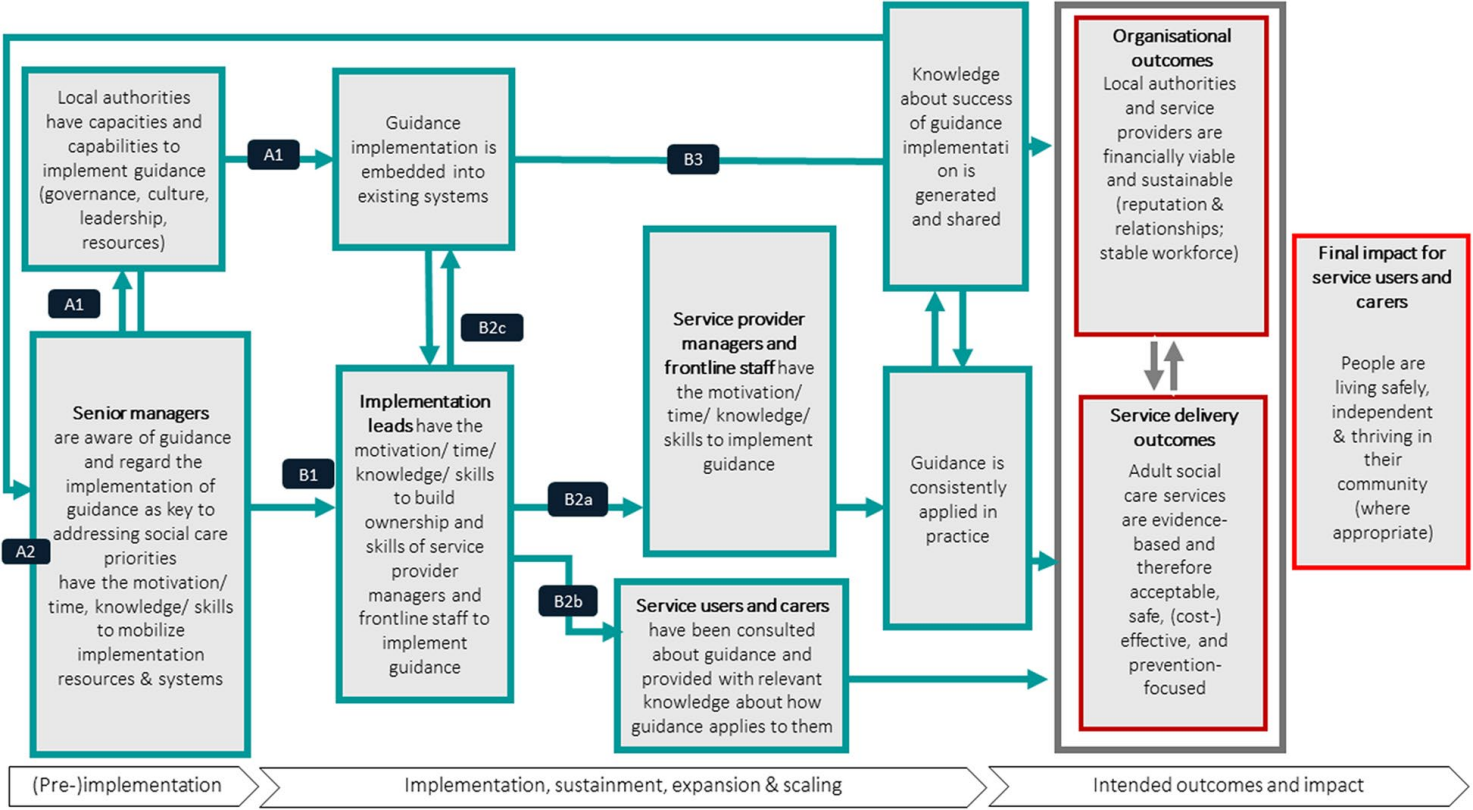
Theory of Change map for implementing guidelines in local authorities

The resulting ToC map developed across the three sites is shown in Fig. 1. Using the backwards-to-forwards logic, we first describe what our participants envisaged to be the intended final impacts of NICE social care guideline implementation, then identify the short- and long-term outcomes they expected would lead to the intended final impacts, and finally detail past, current, or planned implementation activities taken to achieve them. This process revealed some assumptions and uncertainties about how guidelines are implemented and their intended outcomes.

### Final impact

Participants agreed that the final impact goal of implementing guidelines was to increase, where appropriate, the number of adults in need of social care who can live safely and actively in the community.

### Long-term outcomes

Long-term outcomes regarded as contributing to this impact goal can be categorised into service delivery and organisational levels.

At the service delivery level, participants thought guideline implementation could help to achieve services that were high-quality, safe, effective, and good value for money. Given that guideline recommendations for many topic areas represented operationalisations of the prevention-focused national legislation (the 2014 Care Act), their implementation was regarded as supporting a shift from risk- to prevention-focused services, reflected, for example, in a reduction in intrusive and expensive safeguarding services and an increase in lower-intensity support. Participants considered this kind of shift, both over time and at a population-level, to be the only way to be able to continue to afford costly support arrangements where those were needed, and achieve overall financial sustainability.

At the organisational level, participants viewed guideline implementation as important for achieving financial viability, maintaining a stable and satisfied workforce, and building a positive reputation and strong relationships with stakeholders such as staff, service users, carers and partners (e.g., inspection body, local healthcare commissioners or providers, wider public). For example, when coordinating care with health professionals, frontline social care staff could more successfully negotiate that independent living and prevention targets should be considered alongside clinical targets. Referring to guidelines published by the same reputable body that published clinical guidelines was elevating the power of social care professionals relative to healthcare professionals in discussion about a person’s care.

Additionally, implementing guidelines was regarded as contributing to workforce satisfaction and stability goals since frontline staff would feel less stressed and more content with their work knowing they had done everything in line with guidance, especially when the outcome for the person they cared for was not what they hoped for. A stable, competent and satisfied workforce was seen as a means to achieve the above-mentioned service delivery outcomes, creating a positive cycle of organisational sustainability.

### Short- and medium-term outcomes

Participants believed that changes in attitudes, knowledge, skills, and behaviours among managers and frontline staff were necessary to realise the aforementioned long-term outcomes. Such changes would be reflected in the consistent use of guidelines in practice and the communication of the value of guideline implementation at the organisational level, regionally and nationally. The two medium-term outcomes, ‘guidelines consistently applied in practice’ and ‘knowledge about success of guideline implementation generated and shared’, were regarded as closely interlinked because the value of guideline implementation needed to be promoted to sustain and scale implementation efforts.

For frontline staff to consistently refer to standards and recommendations outlined in guidelines, including those focused on prevention, they needed to feel ownership and motivation, as well as have confidence, knowledge, skills, and time. Participants thought that trust needed to be built or restored and certain beliefs ‘demystified’, including the fear among frontline staff of sharing information about current practices, influenced by past experiences where records of their practice might have been used against them.

Changes required support from service provider managers, who also needed to believe in the value of spending time and effort on implementing guidelines, and have the skills, knowledge, and confidence to support frontline staff.

Senior or middle managers at the local authority responsible for coordinating services, supporting both service managers and (senior) frontline staff, needed to know how best to promote guidelines effectively, build trust and confidence. (This referred to the role of what we called above ‘Implementation Support Practitioners’, which included the aforementioned main contacts and sometimes additional individuals with operational management roles.) How their role was allocated to them could differ: it could be a formal part of the job description or come out of a decision made by the groups with quality improvement responsibilities and be part of their career development (Table 1). Individuals taking on those roles needed to have certain characteristics, such as familiarity with NICE guidelines in healthcare, academic qualifications or interests, links to NICE as an organisation (including access to their implementation team and resources), and experience liaising with senior managers as well as frontline staff. They needed support from senior managers and the time and freedom to develop into these roles.

For this, senior managers needed to be convinced of the value of implementing guidelines and know how to mobilise resources and people to enable and support implementation strategically, which could include securing wider organisational and regional support. They needed to see the implementation of guidelines as a solution to achieving organisational goals and solving problems at hand. Senior managers needed to be convinced to give greater authority to frontline staff so that those could implement the guidelines proactively.

Participants agreed that, to achieve guideline implementation at an organisational level, adequate information-sharing, case record, performance and workforce development systems needed to be in place to support staff in the use of guidelines and provide knowledge on progress of implementation uptake, and whether guideline implementation led to changes in practice. This knowledge was necessary to convince senior management of the value of guideline implementation and allowed them to use it to justify investments in implementation towards other stakeholders, such as their governing committees, local or national government, and electorate. Furthermore, participants explained that those systems, if well-designed and including rich data (e.g., on reasons when and why frontline staff were unable to comply with good practice), could provide important knowledge for senior managers to better support ‘Implementation Support Practitioners’ and frontline staff from a strategic level.

Whilst less was mentioned about the role of service users and carers in guideline implementation, there was consensus that representatives needed to be consulted about what information they had about guidelines topics and provide targeted information about the contents of guidelines and how it applied to them. Service users and carers needed to be aware of, or even knowledgeable about, guidelines to understand their rights and feel confident that those rights were upheld in line with guidelines. This needed to be part of a responsive feedback system, in which service users and carers had clarity and transparency about what service or care they could expect to receive and why, as well as the ability to voice concerns and questions. If this was achieved, they could become active in supporting a shift towards a demand-driven, prevention-focused system.

### Implementation activities

#### Phase 1: pre-implementation

A range of activities were pursued to create the conditions for achieving long-term outcomes. First, individuals (usually senior managers) acting as leads for guideline implementation (‘Implementation Support Practitioners’) worked on increasing the organisational readiness for implementing guidelines (A1 and A2). This included activities to establish system capacities and staff capabilities for guideline implementation and wider quality improvement (A1). For example, they created governance structures by establishing teams and job roles, or by incorporating specific guideline implementation responsibilities into existing teams and job roles. They also spent a substantial amount of their time raising awareness of the importance of guidelines within relevant parts of the organisation (i.e., all adult social care and sometimes including children social care) and influencing other senior managers to see the value of its importance (A2). They created support infrastructure (e.g., databases) and ensured that strategic documents and the system incorporated standards into job descriptions. Many of these activities could be conceptualised under ‘*pre-implementation*’ since their aims were to create conditions for guideline implementation. In reality, however, they were often ongoing and happened during implementation processes.

#### Phase 2: implementation, sustainment, expansion and scaling

Given the large number of guidelines and recommendations, senior and middle managers from the groups created above (A1) performed a range of activities to decide which guideline topics or recommendations to prioritise (B1). This prioritisation process commonly took place through a systematic assessment process, during which compliance with guideline recommendations was checked and potential gaps in good practice identified using some form of checklist (the ‘baseline assessments’) provided by NICE on their website. Alternatively, a problem in current service provision was identified first, and then the relevant guidelines identified without aiming to ensure that all guidelines published by NICE had been assessed for relevance and adherence. The group would then allocate responsibilities for implementing guidelines, whilst keeping an eye on how activities progressed, and providing strategic support so that learnings could be utilised for wider scaling of guideline implementation (C).

Operational managers, usually with some supervision from their line manager or a senior manager, led a wide range of different implementation activities involving service providers, service users, carers and local authority support staff. Activities with service provider organisations frequently targeted provider managers and frontline staff, and included auditing, organising training, knowledge exchange and capacity-building events, case mapping and drop-in clinics, practice toolkits and dissemination of information (B2a). These activities commonly had multiple purposes, including gathering information about current practice, educating service providers about guideline recommendations, their legal anchoring (if applicable), and how to apply them in practice. To achieve planned outcomes, implementation activities needed to generate positive feelings towards the use of evidence (e.g., promoting the importance of learning from mistakes) and improve critical and reflective skills to apply guidelines proactively going forward.

Other activities were directly related to implementing specific guideline recommendations that required consulting with and providing information to service users and carers (B2b). These included surveys or meetings with service users and carers to assess their knowledge about relevant guidelines and how they wanted to be informed about guidelines contents, the care they should be receiving, rights and responsibilities. Another set of activities, conducted with support staff, such as IT or human resources, was focused on modifying systems and procedures in line with a specific recommendation (B2c). This included, for example, incorporating guideline-specific contents into workforce development and training programmes or incorporating questions about adherence to a particular standard into case record and audit systems.

The success of, or learning from, the implementation process was captured by operational managers together with other operational or senior managers responsible for managing systems (B3). This included gathering narrative information from case studies to create best practice examples (‘story boards’) or conducting questionnaires that were created to be distributed to frontline staff (or their managers) before and after they participated in the implementation activities (e.g., training). This knowledge was then disseminated inside as well as outside of the organisation, for example by giving presentations at regional meetings with other representatives from local authorities or at national meetings organised by NICE or concerned with sharing knowledge about implementation of guidelines and evidence-based practice (B3).

Sometimes, the learning contributed to the development of a blueprint for guideline implementation that could be shared with other parts of the organisation. Ultimately, activities had purposes to [1] seek further buy-in from senior managers, which was expected to lead to more investment in systems and resources to support guideline implementation; and [2] to promote individuals’ and organisations’ abilities to delivery high-quality care to the national inspection body, regional partners (e.g., NHS) and to service users and carers.

### Assumptions and uncertainties

Overall, there were many uncertainties as to what kind of outcomes could be changed through guideline implementation activities, and how, as there were many organisational and system-wide pressures and constraints that currently prevented successful implementation. This means that the influence of activities on outcomes might be overestimated and much more indirect in reality. Participants illustrated through examples how the logical pathways from outcomes to final impact were strongly influenced by the challenging context in which they operated. For example, the major focus on what we categorised as pre-implementation activities and outcomes was due to the lack of strategic priority and resources given to or available for quality improvement, which meant that those needed first to be created.

### Potential indicators and measurement instruments

Participants agreed that, currently, only limited information is collected to measure changes in adherence to guidelines, or consequences linked to such changes in adherence. However, some local authorities employed questionnaires before and after activities, such as training, to measure changes in knowledge and awareness of guidelines among service provider managers and frontline staff. Workshop participants made several suggestions for the kind of data that could be collected going forward.

With regards to measuring adherence to or coverage of guidelines, participants discussed that possible data sources included assessment forms, case records, referrals statistics, staff surveys, and supervision plans. Participants thought it was important and feasible to measure staff knowledge and confidence concerning the use of guidelines. For service user outcomes, it was important to look at how decision-making points (e.g., initiating a multi-agency assessment) typically connected to outcomes (e.g., safety) and economic consequences (e.g., cost of services) changed before and after applying guidelines, as well as measuring staff perceptions of changes in service users and carers.

In addition to measuring change in impacts related to guidance specifically, participants felt it was important to measure long-term outcomes, such as service user satisfaction and staff turnover, even if these were influenced by a range of factors and not just their efforts to implement guidance. Participants discussed how measuring service users’ and carers’ quality of life and satisfaction was important to inform difficult trade-off decisions between costs and benefits (thus ensuring even costly social care was funded if it was known to lead to substantial quality of life changes for a person).

## Discussion

Using a participatory ToC method, we sought to uncover what stakeholders’ views about how guideline implementation in social care could be implemented at local authority sites and how it might improve long-term outcomes and achieve impact. We identified some necessary implementation conditions and explored how impact and outcomes might be measured. This knowledge can stimulate reflections to inform implementation practice as well as guide the design and evaluation of future implementation strategies.

### Contributions

This study makes two important contributions: First, we gained insights into the implementation activities needed by different groups of individuals to realise desirable outcomes of guideline implementation. This understanding helps refine implementation strategies. Second, the study provides insights into the future evaluation of guideline implementation strategies, including what kind of evaluation questions might be prioritised and how such changes might be measured.

With regards to the first contribution, a wide range of activities were undertaken by ‘Implementation Support Practitioners’ in local authorities to promote the use of the guidelines at different organisational levels as well as externally. These activities likely require a wide range of skills, including relational ones (e.g., persuasion, confidence building), to achieve motivation, trust and ownership. Based on their systematic review of drivers and barriers for implementing guidance in long-term care, McArthur et al. [4] conclude that successful implementation strategies need to focus on changing staff motivation and confidence. Recent implementation research [29, 31, 32] discusses the need for better understanding of the wide range of skills and attributes required by ‘Implementation Support Practitioners’. In the discourse, a greater focus is given to “co-creation or exchange models” of implementation, hypothesising that, by creating dialogue among various stakeholders based on principles of reciprocity, mutuality, and trust, including with practitioners, researchers and end-users, such models are more sustainable in a context of poor infrastructure and resources [33]. More knowledge is needed of how to develop those co-creation or exchange models in social care context, combating the many barriers towards the uptake of evidence-based practice, including inadequate governance structures and resources [4, 34].

Another key implementation activity to achieve envisaged outcomes was sharing success at different levels within and outside the care organisation to convince others of the benefits or value of implementation: this is an important step to increase resources for further and wider implementation [26]. The ways in which social care data systems can be developed and used to continuously improve implementation strategies in practice over time, as recommended in the health literature [35], is not well understood. Overall, whilst our findings resonate with propositions from the literature that implementation strategies need to respond strongly to local context, achieving the envisaged final impact might require broader system changes that are beyond local authorities’ control. This might include expanding NICE’s national dissemination and implementation strategies to support local authorities in implementing guidelines, or increasing the involvement and support from other agencies such as those responsible for workforce development, inspection and regulation.

With regards to our study’s second contribution, our findings underscore the need for a clearer understanding of the alignment of implementation strategies, outcomes, and evaluation measures, as emphasised by Tomasone et al. [20]. It was interesting to note that participants were less concerned about establishing a causal link between their efforts to implement guidelines and final outcomes, which might reflect an awareness of the complexity of the system and many different components that needed to change. Additional work on evaluation methods could prioritise the development or selection of more refined instruments for measuring relational changes among those who are part of the implementation process. New tools have been suggested for measuring trusting relationships as an indicator for successful implementation, including those that measure high-quality communication, empathy-driven exchanges, authenticity and co-learning [36]. Ultimately, there needs to be a process to recognise and, where needed, reconcile different outcomes and economic consequences desired by different groups of people, including the public and service users and carers, whose views might be different from the local authority’s. For example, service users and carers might be more interested in outcomes that reflect meaningful improvements in their experiences of care or changes in their lives [37]. Similarly, there might be other perspectives to be considered, including those of frontline staff, local politicians, inspection and quality assurance representatives, and other agencies providing services such as housing or health. Methods and techniques for effectively identifying and reconciling different stakeholder perspectives are starting to emerge in policy and programme evaluation research [38].

### Strengths and limitations

Finally, in addition to highlighting the contribution of using a ToC method, it is important to reflect on some of the strengths and challenges, many of which have been discussed in the literature [39–41]. We addressed difficulties in engagement, for example, because of participants’ busy schedules, competing priorities, and an initial lack of clarity of the ToC method and its purpose, through strategies such as setting up preparation meetings with individuals or small groups, providing user-friendly and site-specific briefing materials, and by offering flexible ways to provide feedback. The workshops sessions were co-facilitated by a researcher specialised in ToC methods (EB), and extensive expertise in facilitating these workshops in different contexts, and the study’s lead investigator (AB). This helped to address some potential social biases that are common when using qualitative methods. For example, the ToC specialist could focus on clarifying ToC language and terminology, levelling up understanding about the method by all attendees and querying responses to identify common themes, prioritise and seek agreement on. At the same time, the lead investigator could bring in contents from previous conversations with individuals that helped to progress with the ToC during the relatively short session. Most participants appreciated the value of using the ToC approach to think more strategically about guideline implementation. However, our workshop participants represent a small proportion of the overall staff group. It is possible that they might have different characteristics from other staff such as particularly high levels of job satisfaction influencing positive beliefs that they can ‘make a difference’ and change something important within their organisation. Other challenges included getting people together for the purpose of the research and gathering feedback from them in between and after workshops. We sought to address this by adapting the procedures to the communication preferences and routines of the participants and offering flexibilities in how they could provide feedback to the ToC maps. However, it is likely that more could be done to achieve greater levels of participation. Considering the many complexities in managing participatory processes, it would be helpful if future studies that use ToC methods would reflect more on reporting the relational processes.

## Conclusion

Understanding how national guidelines can be implemented and evaluated is an important first step to address the quality-of-care issues that prevail in the long-term care sector internationally. A wide range of activities and skills are required to respond to the challenging condition in which guidelines get implemented and build co-creation models of implementation. Clearer alignment between implementation strategies, outcomes, and evaluation measures and agreement on how to assess relational changes are needed. These insights inform better implementation practices and robust evaluation frameworks for implementing guidelines.

## Electronic supplementary material

Below is the link to the electronic supplementary material.

Supplementary Material 1.

## Data Availability

No datasets were generated or analysed during the current study.

## Abbreviations

NICE: National Institute for Health and Care Excellence
ToC: Theory of Change
