# Supplementary material for: Implementing national care guidelines in local authorities in England and Wales: a theory-of-change
Source: BMC Health Serv Res. 2024 Oct 12;24:1224. doi: 10.1186/s12913-024-11707-4 (PMC11470695; doi:10.1186/s12913-024-11707-4)
Supplement: Supplementary file 1 — Supplementary Material 1. [file 12913_2024_11707_MOESM1_ESM.docx]

**Table 5 Checklist for reporting ToC in Public Health Interventions**

From: Using theory of change to design and evaluate public health interventions: a systematic review (Breuer et al., 2016)

**1. Is the ToC approach defined?**

**a. Is a definition of ToC given by the authors?**

YES

“By applying a backwards-to-forward logic, it draws out the potential causal pathways and multiple interlinks between activities and outcomes, and their relationship to final impact, which are visualised in a graph, the ‘ToC map’”

“a process, in which stakeholders who have knowledge of the topic reach an agreement as to the intended impact of a programme, and how the desired impact can be achieved through a logical sequence of intermediate outcomes”

**b. Do the authors explain their reasons for using a ToC approach?**

YES

“It can contribute to a greater understanding of how to optimise implementation processes, by providing knowledge of factors likely to influence outcomes, as well as inform the choice of outcome measure for an evaluation. The approach is increasing accepted to be used alongside implementation theories, models, and frameworks.”

“Because it does not require pre-existing evidence from the literature, and is regarded a valuable the process by stakeholders (as it supports their strategic planning), it is very suitable for use in social care practice”

**2. Is the ToC development process described?**

**a. Are the methods used to develop the ToC, such as stakeholder meetings and interviews, document reviews, programme observation, existing conceptual frameworks or published research, described?**

YES – p4

**b. Where stakeholders are involved, is it clear how many stakeholders participated, what their role is in relation to the intervention, how they were consulted (e.g. number of interviews, focus groups, ToC workshops) and the extent to which the consultations were participatory?**

YES – p4

**c. Is the method used to compile the data into a ToC described? (including how disagreements between stakeholders were resolved)**

YES – p7 “Conflicts or disagreement were resolved through open and respectful communication, in which all perspectives were first validated and then responses were brought back to the purpose of the workshop before deciding about group priorities and relevance to the ToC map.”

**d. Is the extent to which stakeholders were able to validate the resultant ToC and were owners of the final product described?**

YES – p8 “After the first round of workshops, the ToC maps were developed further by the researchers, presented to the participants in the second workshops and then iteratively refined by the researchers using additional data from one-on-one or group meetings.”

**3. Is the resultant ToC (or a summary thereof) depicted in a diagrammatic form and does it include?**

a. The long-term outcome or impact of the intervention

YES -p9

b. The anticipated short and medium term outcomes and the process of change

YES – P10/11

**c. The intervention components which happen at different stages of the pathway**

YES – Activities described p11-14

d. The context of the intervention

YES – p14

e. Assumptions about how change would occur

YES - p14

f. Additional ToC elements such as indicators, supporting research evidence, beneficiaries, actors in the context, sphere of influence and timelines where relevant.

YES – p14-15

**4. Is the process of intervention development from the ToC described?**

a. Are the methods of how interventions were refined from the ToC to something which can be implemented described? (For example, further stakeholder workshops, interviews, systematic literature reviews)

PARTLY - Not part of the study as such but considered discussion section

**5. Is the way in which the ToC was used to develop and implement the evaluation described?**

a. Are evaluation research questions generated from the ToC?

PARTLY - Not part of the study as such but considered discussion section

b. Is the role of ToC in the design, plan or conduct of the evaluation clear?

PARTLY - Not part of the study as such but considered discussion section

c. Does the paper describe the extent to which the key elements described in the ToC were measured in the evaluation (i.e. impact, short and medium term outcomes and the process of change, context, assumptions and the intervention)?

NO – Not applicable

d. Does the paper describe whether and how process indicators were used to improve the quality of the intervention?

NO – Not applicable

e. Is the role of the ToC in the analysis of the results of the evaluation clear?

NO – Not applicable

f. Is the role of ToC in the interpretation of the results of the evaluation described? (including the breakdown of programme theory, unanticipated outcomes and causation including the strength and direction of causal relationships)

NO - Not applicable

Breuer, E., Lee, L., De Silva, M., & Lund, C. (2016). Using theory of change to design and evaluate public health interventions: a systematic review. *Implement Sci*, *11*, 63. <https://doi.org/10.1186/s13012-016-0422-6>
